# Association between tea consumption and prevention of coronary artery disease: A systematic review and dose-response meta-analysis

**DOI:** 10.3389/fnut.2022.1021405

**Published:** 2022-11-24

**Authors:** Xin Yang, Haiyun Dai, Ruihang Deng, Ziang Zhang, Yiwen Quan, Mohan Giri, Jian Shen

**Affiliations:** ^1^The First College of Clinical Medicine, Chongqing Medical University, Chongqing, China; ^2^Department of Respiratory and Critical Care Medicine, The First Affiliated Hospital of Chongqing Medical University, Chongqing, China; ^3^Department of Cardiology, The First Affiliated Hospital of Chongqing Medical University, Chongqing, China

**Keywords:** tea, coronary artery disease, cardiovascular disease, dose-response, meta-analysis

## Abstract

**Background:**

Evidence from previous studies reporting on the relationship between tea consumption and its preventive effect on coronary artery disease (CAD) has conflicting outcomes. With the accumulation of new clinical evidence, we conducted this meta-analysis to assess tea consumption and CAD risk.

**Methods:**

We searched PubMed, EMBASE, Cochrane Library, and Medline databases for published observational studies from their inception to May 2022. A random-effects model was used to calculate risk ratios with 95% confidence intervals. We also conducted linear and non-linear dose-response meta-analyses to analyze the association. We regarded that one cup equals 237 mL. Subgroup analyses and univariate meta-regression were conducted to explore the source of heterogeneity.

**Results:**

A total of 35 studies, including 24 on green tea and 11 on black tea consumption, were included in this meta-analysis. An inverse association for the risk of CAD was observed for black tea (RR: 0.85; 95% CI: 0.76, 0.96) and green tea (RR: 0.93; 95% CI: 0.88, 0.99). The dose-response meta-analysis showed that drinking less than four cups of black tea daily may effectively prevent CAD, while more than 4–6 cups/d will promote disease risk. Furthermore, the dose-response relationship between green tea consumption and the prevention of CAD showed that the risk of CAD gradually decreased as green tea consumption increased. We also demonstrated that the more cups of green tea consumed, the lower the risk of CAD. In the subgroup analysis by continent, a significant negative correlation between CAD risk and green tea consumption was observed in the Asian population (RR: 0.92; 95% CI: 0.85, 0.99) but not in the western population [North America (RR: 0.97; 95% CI: 0.92, 1.03), Europe/Oceana (RR: 0.91; 95% CI: 0.78, 1.07)].

**Conclusions:**

Higher green tea consumption was associated with reduced CAD risk, but drinking more than 4–6 cups of black tea per day may increase the risk. This study offers new insight into the relationship between tea consumption and its preventive effect on CAD. However, further large prospective cohort studies are needed to validate these findings.

**Systematic review registration:**

The protocol of this systematic review was registered in the International Prospective Register of Systematic Reviews (PROSPERO) system (CRD42022348069).

## Introduction

Despite significant advances in prevention and treatment, coronary artery disease (CAD) arising from atherosclerosis is a leading cause of morbidity, mortality, and disability worldwide (1, 2). With around 11.39 million CAD patients, the prevalence of CAD is increasing in China (3). The detrimental effect of CAD has become one of the key issues in terms of the economic burden on people in both developed and developing countries (4). Therefore, the primary prevention of CAD has become one of the major focuses of public health and preventive medicine.

As one of the most popular beverages in the world, tea consumption (black or green) has been regarded as health-promoting for millennia, and the polyphenols, particularly flavonoids in tea, are recognized as natural antioxidants and play an essential role in preventing cardiovascular disease, mainly due to their antiatherogenic and antithrombotic properties (5–7). Generally, tea may be classified into six types depending on its processing and fermentation: green tea, black tea, white tea, yellow tea, oolong tea, and dark tea (8, 9). Tea drinking and its preventative impact on CAD have previously been studied (10–17). The findings of these studies, however, are inconsistent and conflicting. Previous studies have shown that drinking tea may lower the risk of morbidity and mortality from cardiovascular disease (10–12). However, some studies have shown that drinking tea has minimal effect on cardiovascular disease (13, 15, 17). Wang et al. (18) conducted a meta-analysis that examined the relationship between black tea intake, green tea consumption, and the risk of CAD using published data from 18 studies. They revealed that black tea drinking was not associated with a lower risk of CAD, whereas green tea consumption reduced the risk of CAD. This meta-analysis, however, did not examine the dose-response association between tea drinking and the risk of CAD in detail. Furthermore, the studies in their meta-analysis varied greatly due to the types of tea, the differences in the surveyed population, gender, age, and other confounding factors.

To date, the association between tea consumption and the risk of CAD has not yet been fully elucidated. In this study, we performed a meta-analysis to provide comprehensive and updated evidence on the relationship between tea consumption and its preventive effect on CAD. Additionally, we conducted a dose-response meta-analysis to determine the optimal tea consumption for preventing CAD.

## Methods

The protocol of this systematic review was registered in the International Prospective Register of Systematic Reviews (PROSPERO) system (CRD42022348069). For this systematic review and meta-analysis, we followed the preferred reporting items for systematic reviews and meta-analyses (PRISMA) Guidelines (19). For the meta-analysis, the population, intervention, comparison, and outcome (PICO) format was used, i.e., P: people without coronary heart disease; I: green tea OR black tea; C: don't drink tea; O: the mortality or incidence of coronary heart disease.

### Search strategy

We conducted a comprehensive search on the PubMed, Embase, Medline, and Cochrane library databases from their inception to May 2022 for prospective cohort studies published in journals that described the association between drinking different types of tea and the risk of CAD. The MeSH and search terms were: “tea” OR “black tea” OR “green tea” OR “flavonoid” OR “catechin” OR “cyanidanol” OR “theaflavin” combined with “coronary Disease” OR “Myocardial Ischemia” OR “myocard^*^ infarct^*^” OR “coronary disease^*^” OR “ischemic heart” OR “atherosclerosis” OR “atherosclero^*^” OR “angina^*^” The search was restricted to human research, and the language of the publication was limited to English. We also reviewed the references of the retrieved articles to identify additional studies. One investigator (XY) screened the titles and abstracts of all identified articles; two researchers (XY and HD) read the full text to assess the eligible studies.

### Eligibility criteria for study inclusion

This meta-analysis included studies that met the following criteria: (a) Study type: a cohort study or case-control study; (b) the exposure of interest: tea consumption; (c) the outcome: the prevalence or mortality of CAD (including myocardial infarction, CAD, non-stroke cardiovascular disease and other coronary events), (d) Studies that reported the 95% confidence interval odds ratio (OR) or relative risk (RR) of the relationship between black tea or green tea and CAD or provided other corresponding data to calculate the variance. If duplicate publications from the same study were found, we used the results of the study with the largest number of cases. The following were the exclusion criteria: meta-analyses, reviews, duplicate studies, and studies lacking adequate data.

### Data extraction

The two researchers (XY and ZZ) independently extracted data using a standardized electronic format. The following data were extracted for each eligible study: the first author's name, year of publication, study site, sample size, sex, age range or average age, follow-up time, number of cases, exposure assessment methods, outcome measurements, odds ratio (OR) or relative risk (RR) between black and green tea and CAD and corresponding 95%CI. For dose-response analysis, we standardized all data as cup/day when the study reported daily, weekly, or monthly doses or times. When the tea consumption metric is expressed in grams (g) or milliliters (ml), we regarded 125 g/month as 2 cups/day and 237 ml as 1 cup (20). The discrepancies in data extraction among the three researchers were solved by a discussion with the third researcher (HD).

### Quality assessment

The methodological quality was evaluated by the Newcastle-Ottawa scale (NOS) (21). NOS is a comprehensive tool that has been validated to assess the quality of observational research in the meta-analysis, and it is based on the following three subscales: selection (4 items), comparability (1 item), and results (3 items). The NOS star system was used to assess the quality of the included studies (range, 0–9 stars). Studies with scores >6 were regarded as high-quality, while those below four were excluded. The quality evaluation was carried out by two authors (ZZ and YQ) independently, and the information was reviewed and verified by another author (XY).

### Statistical analysis

The random effect model was used to calculate the aggregate RR and corresponding 95% CI of the highest and lowest levels of black and green tea consumption and dose-response analysis. Analysis was performed using the Mantel-Haenszel random effects model (22). For studies that include data from multiple queues, we regard the analysis of each queue as an independent report. Seven studies reported estimates of black and green tea consumption and CAD risk by sex (male and female). One article may be divided into two separate reports. In any study that separately expressed lethal CAD and non-lethal MI, the analysis of each gender or subtype of CAD was also considered an independent report. In addition, we also carried out subgroup analyses, such as gender, age, race, etc., to analyze the differences in the role of tea drinking in different populations. For dose-response analysis, we used the methods described by Greenland and Longnecker to calculate trends from the correlation estimates of the relative risk of black and green tea consumption (23). According to this method, tea consumption, the number of cases, RRs, and 95% CI were extracted. We evaluated heterogeneity by estimating the variance between studies using Q and *I*^2^ statistics. In order to adjust the type I and type II errors, we set the significance level to the traditional 0.05. When statistical heterogeneity was detected, the source of heterogeneity was explored, and subgroup analysis and regression were performed. A funnel chart and Egger's test were used to evaluate potential publication bias (24, 25). The trim and fill method was used to examine the possible effect of publication bias on the results (26). All analyses were conducted with STATA 17 software (STATA Corp, College Station, TX). *P*-values were two-tailed, and *p* < 0.05 were considered statistically significant.

## Results

### Search results

**Figure 1:**
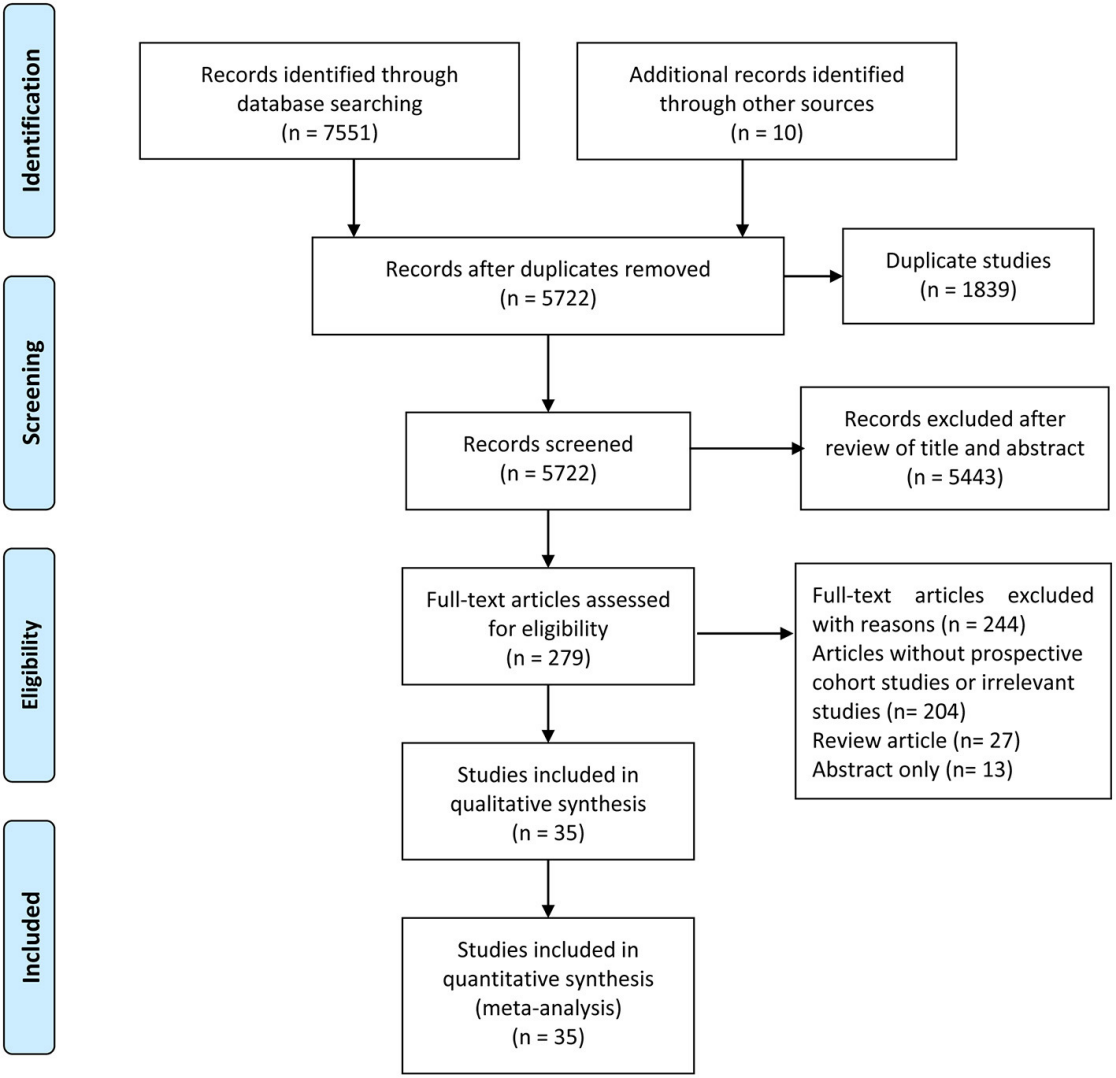
Flow diagram of literature search and study selection.

The flowchart of the literature search and study selection process is presented in Figure 1. We identified 7,551 studies from a database search and ten additional records through other sources, from which 1,839 duplicate studies were excluded. After duplicates were removed, the titles and abstracts of 5,722 records were assessed. A total of 279 full texts were assessed, and after a further detailed evaluation of the full text, 35 prospective observational studies were included in this meta-analysis. Details of reasons for exclusion are reported in Figure 1.

### Study characteristics

The characteristics of the included studies are shown in Table 1 (black tea) and Table 2 (green tea). These studies were published between 1972 and 2020, with follow-up periods ranging from 1 to 34 years. There were 11 studies on black tea (10, 27–36) and 24 studies (13–16, 37–56) on green tea. There were 13 studies from the United States, seven from Europe, and 15 from Asia (China and Japan).

**Table 1:** Epidemiological studies on black tea consumption in association with coronary artery disease (CAD) risk.

**Study (year)**	**Country**	**Design**	**Study period**	**Follow–up**	**Gender**	**Outcome**	**Simple size^2^**	**Tea** **consumption**	**RR/OR (95% CI)**	**Continent**
Stensvold et al. (27)	Norway	Cohort	1976–1988	13 Y	M	CD	141/9,863	<1 cup/d ≥1 cup/d	1 0.49 (0.30–0.81)	Northern Europe
					F	CD	18/10,224	<1 cup/d ≥1 cup/d	1 0.29 (0.07–1.28)	Northern Europe
De Koning Gans et al. (10)	USA	Cohort	1998–2010	13 Y	both	CHD	1,387/37,514	<1 cup/d 1–2 cups/d 2.1–3 cups/d 3.1–4 cups/d 4.1–6 cups/d >6 cups/d	1 0.93 (0.81–1.06) 0.74 (0.72–1.06) 0.88 (0.72–1.06) 0.91 (0.74–1.11) 0.64 (0.46–0.90)	North America
Geleijnset al. (28)	USA	Cohort	1990–2001	12 y	Both	MI	146/4,661	0 1–375 ml/d >375 ml/d	1 0.72 (0.49–1.13) 0.51 (0.30–0.84)	North America
Hertog et al. (29)	Netherland	Cohort	1960–1993	34 y	M	CHD death	43/762	≤ 1 cup/d 1–2 cups/d ≥3 cup/d	1 0.48 (0.22–1.01) 0.55 (0.27 1.13)	Europe
Hertog et al. (30)	USA	Cohort	1983–1996	13 y	M	IHD death	131/1,769	0–300 ml/d 450–750 ml/d 900–1,200 ml/d >1,200 ml/d	1 1.6 (0.80–3.00) 2.1 (1.10–4.10) 2.2 (1.00–4.70)	North America
Keli et al. (31)	Netherlands	Cohort	1970–1996	27 y	M	stroke	42/510	<330 ml/d 330–586.6 ml/d ≥586.7 ml/d	1 0.6 (0.31–1.18) 0.38 (0.15–0.99)	Europe
Sesso et al. (32)	USA	Case control	1984–1999	16 y	Both	MI	341/680	0 1–3 cup/month 1–6 cup/week ≥1 cup/d	1 0.86 (0.70–1.04) 0.86 (0.70–1.06) 0.75 (0.61 0.92)	North America
Woodward et al. (33)	UK	Cause control	1984–1993	7.7 y	F	IHD	47/6,703	0 cup/day 1–2 cups/day 3–4 cups/day 5 cups/day	1 0.74 (0.25–2.15) 0.76 (0.25–2.25) 1.41 (0.54–3.69)	Europe
					M	IHD	159/5,724	0 cup/day 1–2 cups/day 3–4 cups/day 5 cups/day	1 1.17 (0.6–2.29) 1.34 (0.71–2.53) 1.96 (1.07–3.57)	Europe
Rosenberg et al. (34)	USA	Cause control	1976–1979	3 y	F	MI	472/1,423	0 cup/day 1–2 cups/day 3–4 cups/day 5 cups/day	1 0.81 (0.69–0.97) 0.94 (0.71–1.25)	North America
Klatsky et al. (35)	USA	Cause control	1978–1985	7 y	both	MI	4208/125356	0 cup/day <1 cup/day 1–3 cups/day >=4 cups/day	1 0.77 (0.72–0.83) 0.92 (0.85–1) 1.08 (0.91–1.29)	North America
Sesso et al. (36)	USA	Cohort	1977–1988	11 y	both	CHD	1,613/1,7228	0 cup/day <1 cup/d 1cup/d 2 cups/d 3 cups/d ≥4 cups/d	1 0.97 (0.85–1.11) 0.98 (0.89–1.09) 0.93 (0.83–1.04) 0.85 (0.69–1.06) 0.98 (0.82–1.18)	North America

CD, coronary death; CHD, coronary heart disease; MI, myocardial infarction; CAD, coronary artery disease; d, day; RR, relative risk; OR, odd ratio; CI, confidence interval.

2 Cases/controls or cohort size.

**Table 2:** Epidemiological studies on green tea consumption in association with coronary artery disease (CAD) risk.

**Study (year)**	**Country**	**Design**	**Study period**	**Follow–up**	**Gender**	**Outcome**	**Simple size^2^**	**Tea** **consumption**	**RR/OR, (95% CI)**	**Continent**
Tavani et al. (37)	Italy	Case control	1995–1999	4 y	Both	MI	507/985	<1 cup/d ≥1 cup/d	1 1 (0.7–1.3)	Europe
Thrift et al. (38)	Australia	Case control	1990–1992	3 y	Both	Stroke	331/662	<1 cup/day ≥1 cup/day	1 1.35 (0.93–1.96)	Europe
Tian et al. (39)	China	Cohort	2008–2013	5 y	M	CHD	968/8,585	<1 cup/day ≥1 cup/day	1 0.84 (0.74–0.95)	Asia
					F	CHD	1,097/9,789	<1 cup/day ≥1 cup/day	1 0.83 (0.72–0.96)	Asia
Wang et al. (40)	China	Case control	2008–2009	1 y	M	CAD	246/379	0 g/month <125 g/month 125–249 g/month >250 g/month	1 1 (0.59–1.72) 0.45 (0.24–0.82) 0.42 (0.22–0.81)	Asia
					F	CAD	89/151	<1 cup/day ≥1 cup/day	1 0.65 (0.27–1.57)	Asia
Hirvonen et al. (41)	USA	Cohort	1986–1993	6 y	F	CD	815/27,122	<1 cup/day ≥1 cup/day	1 1.09 (0.92–1.30)	North America
Wen et al. (42)	China	Case control	2002–2006	4 y	M	MI	518/2,590	<1 cup/day ≥1 cup/day	1 0.89 (0.72–1.09)	Asia
Sato et al. (15)	Japan	Cohort	1984–1988	4 y	F	Stroke	174/9,511	<5 cups/day ≥5 cups/day	1 0.35 (0.24–0.51)	Asia
Hirano et al. (43)	USA	Cohort	1986–1993	6 y	F	CD	815/24,304	<1 cup/day ≥1 cup/day	m 1 1.09 (0.91–1.30)	North America
Gramenzi et al. (14)	Italy	Case control	1983–1989	5 y	F	MI	287/936	<1 cup/day 1 cup/day >1 cups/day	1 0.66 (0.47–0.91) 0.88 (0.57–1.37)	Europe
Klatsky et al. (44)	USA	Cohort	1978–1986	5 y	both	MI	740/11,900	0 cup/day <1 cup/day 1–3 cups/day 4–6 cups/day >6 cups/day	1 0.82 (0.7–0.98) 1.01 (0.83–1.22) 0.79 (0.44–1.42) 1.49 (0.77–2.89)	North America
Boston study et al. (45)	USA	Case control			both	MI	276/1,380	0 cup/day 1–5 cups/day ≥6 cups/day	1 0.9 (0.72–1.11) 0.71 (0.4–1.25)	North America
Hao et al. (46)	China	Case cohort	1999–2003	4 y	both	MI	3,039/6,146	0 cup/day 1 cup/day 2 cups/day 3 cups/day ≥4 cups/day	1 1.1 (1.02–1.18) 1.03 (0.96–1.12) 1.13 (1.05–1.22) 1.15(1.06–1.24)	Asia
Sano et al. (47)	Japan	Case control	1997–2003	6 y	both	CAD	109/203	0–3 cups/day 4–7 cups/day 8–11 cups/day 12–15 cups/day 16–19 cups/day 20–23 cups/day 24–27 cups/day	1 0.79 (0.61–1.03) 0.26 (0.11–0.58) 0.75 (0.33–1.68) 1.12 (0.5–2.53) 0.25 (0.02–3.14) 0.37 (0.03–4.14)	Asia
Miller et al. (48)	USA	Cohort	2000–2013	11.1 y	both	MI	3,115/6,187	0 cup/day <1 cup/day 1 cup/day	1 1.07 (0.89–1.28) 1.04 (0.88–1.22)	North America
Li et al. (49)	China	Cohort	2004–2013	7.2y	both	IHD	6,377/1,28280	0 0–1cup/day 1–1.5 cup/day 1.5–2.5 cups/day >2.5 cups/day	1 0.97 (0.94–1) 0.94 (0.9–0.99) 0.93 (0.87–1) 0.96 (0.9–1.02)	Asia
Kishimoto et al. (50)	Japan	Case control	2008–2017	9 y	both	MI	388/612	<1 cup/day 1–3 cups/day >3 cups/day	1 0.9 (0.79–1.04) 0.82 (0.68–0.98)	Asia
Kokubo et al. (51)	USA	Cohort	1995–2007	13 y	both	CHD	910/82,369	None 1–2 cup/week 3–6 cup/week 1 cup/d 2–3 cups/d >=4 cups/d	1 0.8 (0.61–1.04) 0.99 (0.76–1.29) 0.91 (0.7–1.18) 0.82 (0.67–1.02) 0.99 (0.83–1.2)	North America
Pang et al. (52)	China	Case control	2012–2014	3 y	both	CHD	370/628	0 1–2 cups/d 3 cups/d	1 0.95 (0.83–1.09) 0.7 (0.57–0.86)	Asia
Yan et al. (53)	USA	Cohort	1971–2017	47y	both	cardiovascular disease	250/11,808	0 cup/week 1–7 cup/week 8–14 cup/week >14 cup/week	1 1.17 (0.865–1.577) 1.26 (0.88–1.80) 0.94 (0.58–1.53)	North America
Liu et al. (54)	China	Cohort	1990–2016	27 y	M	cardiovascular disease	11,839/1,62,681	0 ≤ 5 cup/d 5–10 cup/d >10 cup/d	1 0.93 (0.85–1.01) 0.91 (0.85–0.98) 0.86 (0.79–0.93)	Asia
Xiang et al. (55)	China	Case control	2013–2014	1 y	M	CHD	172/277	0 1–2 cups/d 3–5 cups/day >5 cups/d	1 1.11 (0.78–1.56) 1.29 (1.02–1.64) 1.19 (0.93–1.53)	Asia
					F	CHD	94/251	0 1–2 cups/d 3–5 cups/day >5 cups/d	1 1.51 (0.96–2.38) 0.64 (0.39–1.06) 0.79 (0.4–1.58)	Asia
Mineharu et al. (13)	Japan	Cohort	1988–2003	13.1 y	Both	CHD	729/74,789	None 0–1 cup/d 1–2 cups/day 3–5 cups/day >6 cups/day	1 0.34 (0.06–1.75) 0.28 (0.07–1.11) 0.39 (0.18–0.85) 0.42 (0.15–0.92)	Asia
Kuriyama et al. (16)	China	Cohort	1994–2006	13 y	M	CHD	129/19,060	1 cups/d 1–2 cups/d 3–4 cups/d >5 cups/d	1 1.03 (0.62–1.71) 0.96 (0.57–1.62) 0.91 (0.56–1.48)	Asia
					F	CHD	80/21,470	1 cups/d 1–2 cups/d 3–4 cups/d >5 cups/d	1 1.04 (0.54–2.01) 0.79 (0.4–1.56) 0.77 (0.42–1.44)	Asia
Chen et al. (56)	China	Cohort	2013–2014	2 y	F	CHD	157/256	0 1–2 cups/d >3 cups/d	1 1.867 (1.018–3.426) 1.834 (0.947–3.551)	Asia
					M	CHD	91/212	0 1–2 cups/d >3 cups/d	1 0.323 (0.173–0.601) 1.497 (0.436–2.439)	Asia

CD, coronary death; CHD, Coronary Heart Disease; MI, myocardial infarction; CAD, coronary atherosclerotic heart disease; d, day; RR, relative risk; OR, odd ratio; CI, confidence interval.

2 Cases/controls or cohort size.

### Quality of included studies

**Figure 2:**
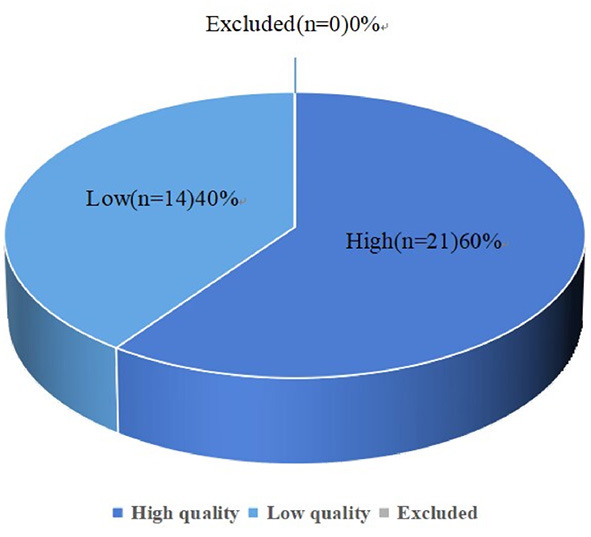
Pie chart showing the results of the Newcastle-Ottawa Scale (NOS) scale.

The overall NOS score of each included study is presented in Supplementary Table 1. Overall, 21 studies were evaluated as having high methodological quality, while the remaining 14 were considered low methodological quality (Figure 2).

### Black tea

**Figure 3:**
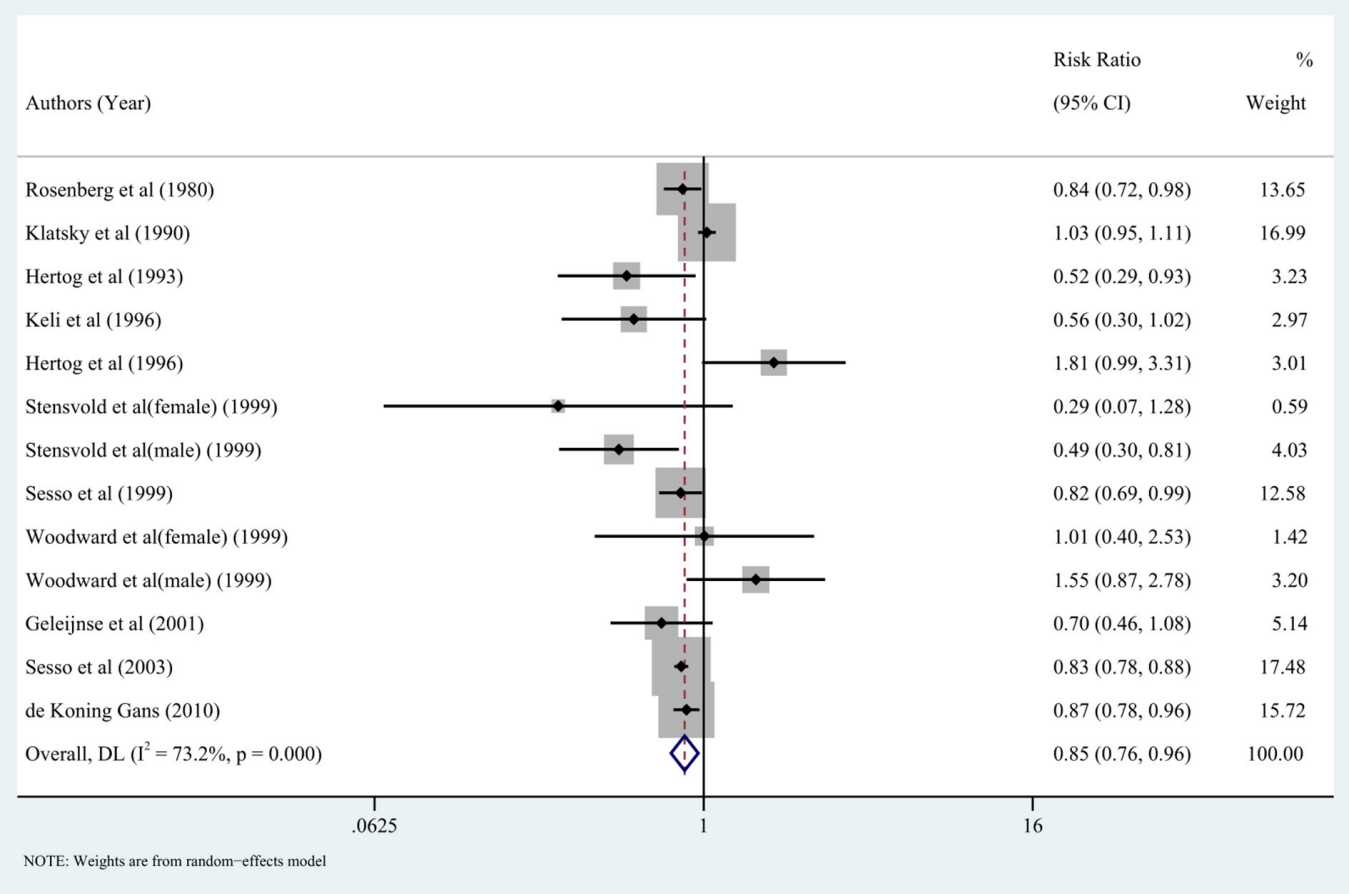
Forest plot: Summary relative risks (RRs) of coronary artery disease (CAD) for comparing the highest black tea consumption with the lowest black tea consumption. The squares indicate study-specific risk estimates (the size of the square reflects the study's statistical weight), the horizontal lines indicate the 95% confidence intervals (CIs), and the diamond shows the summary RR estimate with its corresponding 95% CI.

**Figure 4:**
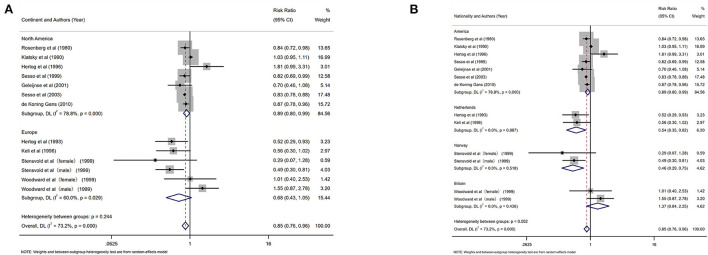
Forest plot: results of subgroup analysis of black tea consumption and coronary artery disease (CAD) risk. **(A)** Forest plot of black tea consumption and CAD risk after subgroup analysis stratified by continent. **(B)** Forest plot of black tea consumption and CAD risk after subgroup analysis stratified by country.

**Figure 5:**
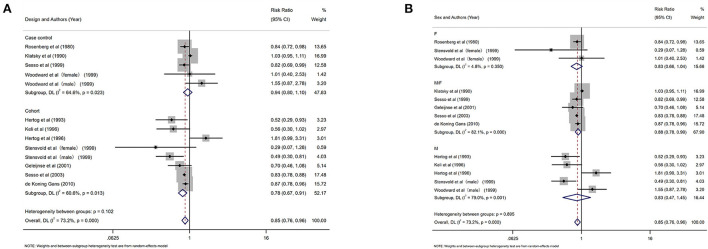
Subgroup analysis of black tea intake and coronary artery disease (CAD) risk. **(A)** Forest plot of subgroup analysis stratified by the study design. **(B)** Forest plot of subgroup analysis stratified by gender.

Eleven studies investigated the relationship between the highest vs. the lowest categories of black tea consumption and the risk of CAD. Figure 3 depicts the RRs of CAD for the highest vs. lowest total black tea consumption categories, demonstrating an association between the highest black tea consumption and CAD risk (summary RR: 0.85; 95% CI: 0.76, 0.96) with a high degree of heterogeneity (*I*^2^ = 73.2%; *P* < 0.001). We performed subgroup and regression analyses to investigate the sources of heterogeneity. Subgroup analyses were performed according to continent, nationality, study design, and gender (Figures 4, 5). Although no source of heterogeneity was found in the subgroup analysis, we found that studies in the United States, Norway, and the Netherlands supported that black tea drinking could reduce the risk of CAD. Notably, sub-group analysis by nationality revealed that black tea might reduce the incidence of CAD by 50% in Norway (RR: 0.46 95% CI:0.29, 0.75) and the Netherlands (RR: 0.54 95% CI: 0.35, 0.82) (Figure 4).

**Figure 6:**
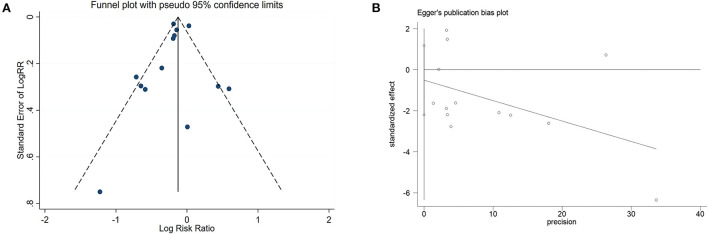
Funnel plot and Egger's test analyses to detect publication bias of studies on black tea consumption and coronary artery disease (CAD) risk. **(A)** Funnel plot: used to detect the publication bias studies. **(B)** Egger chart: used to detect the publication bias of studies.

In addition, we performed a meta-regression analysis on the publication year and follow-up time to check whether the heterogeneity was caused by this (tau = 0.01662), a decrease of 0.00208 compared with the prior (tau 0.0187), indicating that this was attributable to study year differences. The difference in follow-up time could explain 11.12% of the heterogeneity. We created a funnel plot to evaluate the publication bias (Figure 6). We also used Egger's test to assess publication bias quantitatively (Figure 6). Egger's test revealed no evidence of publication bias (*P* = 0.519, 95% CI = −2.20, 1.18).

**Figure 7:**
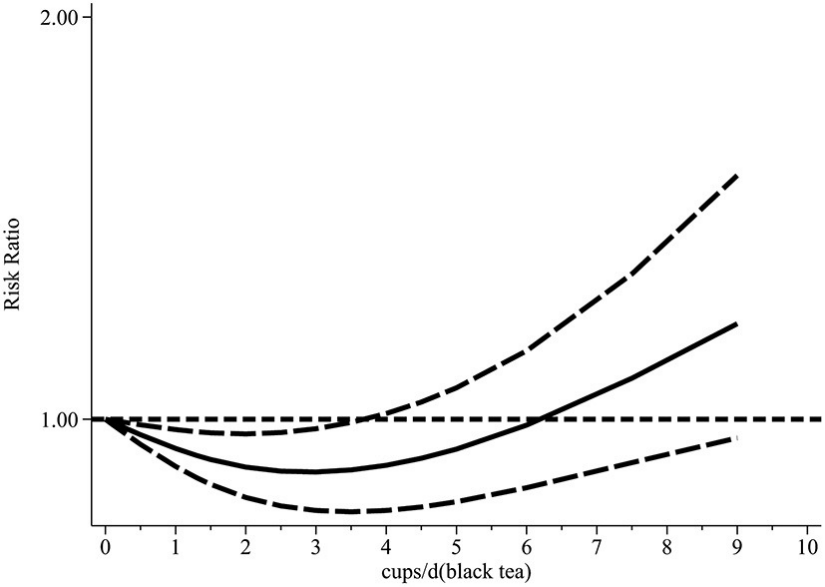
Dose-response relationship between black tea cup number (cups/d) and coronary artery disease (non-linear model).

Our dose-response analysis did not show a linear-dose-response relationship (chi2 (1) = 16.59 Prob>chi2 = 0.0000). Therefore, we established a regression model using the regression coefficients of the nonlinear regression equation and plotted the non-linear dose-response meta-analysis graph. As shown in Figure 7, drinking <3 cups of black tea daily may effectively prevent CAD, while more than 4–6 cups/d will promote the risk of disease.

### Green tea

**Figure 8:**
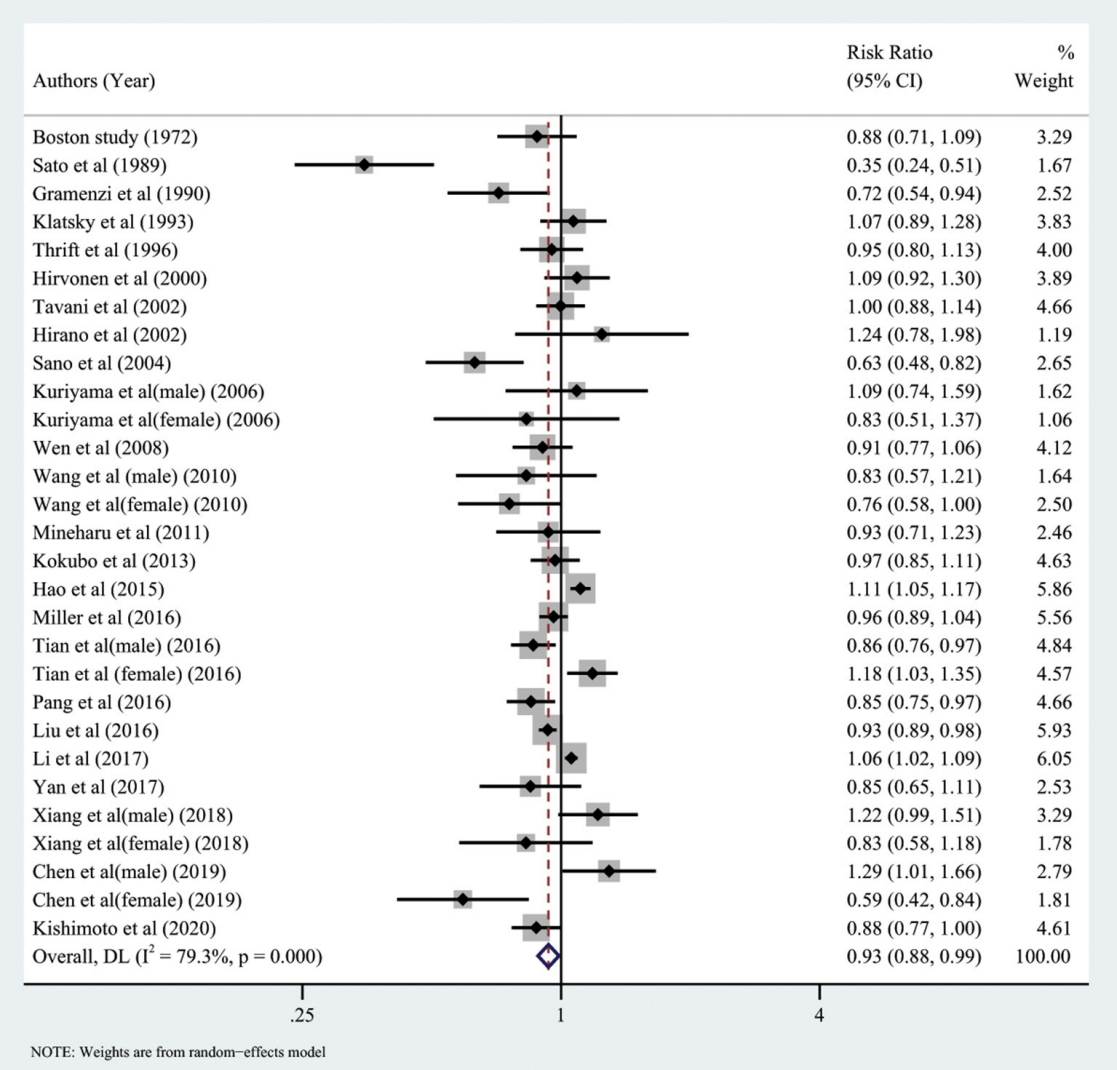
Forest plot: Summary relative risks (RRs) of coronary artery disease for comparing the highest green tea consumption with the lowest green tea consumption. The squares indicate study-specific risk estimates (the size of the square reflects the study's statistical weight), the horizontal lines indicate the 95% confidence intervals (CIs), and the diamond shows the summary RR estimate with its corresponding 95% CI.

**Figure 9:**
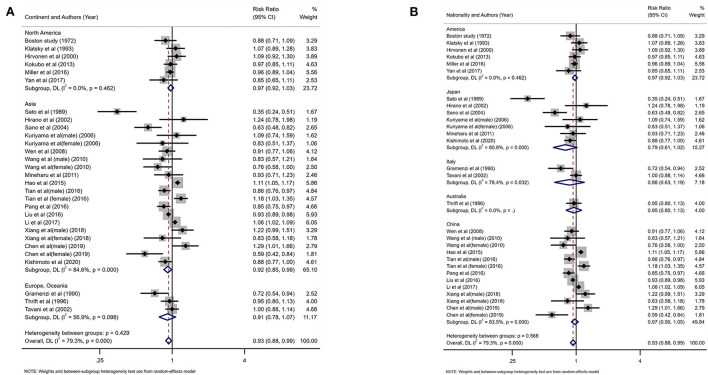
Forest plot: results of subgroup analysis of green tea consumption and coronary artery disease (CAD) risk. **(A)** Forest plot depicting green tea consumption and CAD risk after subgroup analysis stratified by continent. **(B)** Forest plot showing green tea consumption and CAD risk after subgroup analysis stratified based on country.

**Figure 10:**
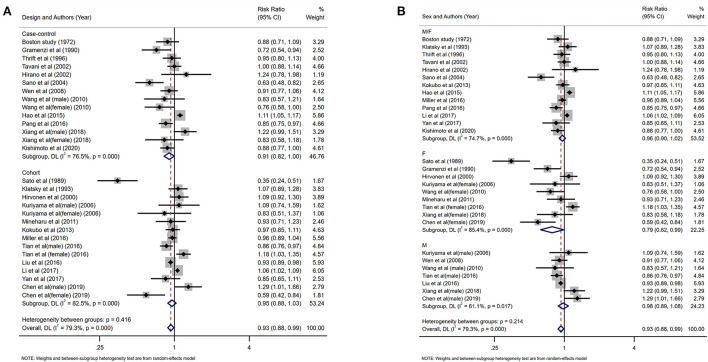
Forest plot depicting the subgroup analysis of green tea intake and coronary artery disease (CAD) risk. **(A)** Subgroup analysis forest plot stratified by the study design. **(B)** Forest plot of subgroup analysis based on gender.

Twenty-four studies investigated the relationship between the highest vs. the lowest categories of green tea consumption and CAD risk. The pooled results showed the association between highest green tea consumption and CAD risk (summary RR: 0.93; 95% CI: 0.88, 0.99), with a high heterogeneity (I^2^ = 79.3%; *P* < 0.001) (Figure 8). We used subgroup and regression analyses to account for the heterogeneity. We also did subgroup analysis according to gender, continent, nationality, study design, and gender (Figures 9, 10). The findings revealed that heterogeneity was associated with nationality: the Japanese group (*I*^2^ = 80.8%, *p* < 0.001), the Italian group (I^2^ = 78.4%, *p* = 0.032), and the Chinese group (*I*^2^ = 83.5%, *p* < 0.001). Further assessment of a meta-analysis for the dimensions of published time and study location showed the heterogeneity of this study (tau^2^ = 0.2892) was higher than the previous (tau^2^ = 0.0134), which indicated that the heterogeneity could not be explained by different years and regional dimensions of the study.

**Figure 11:**
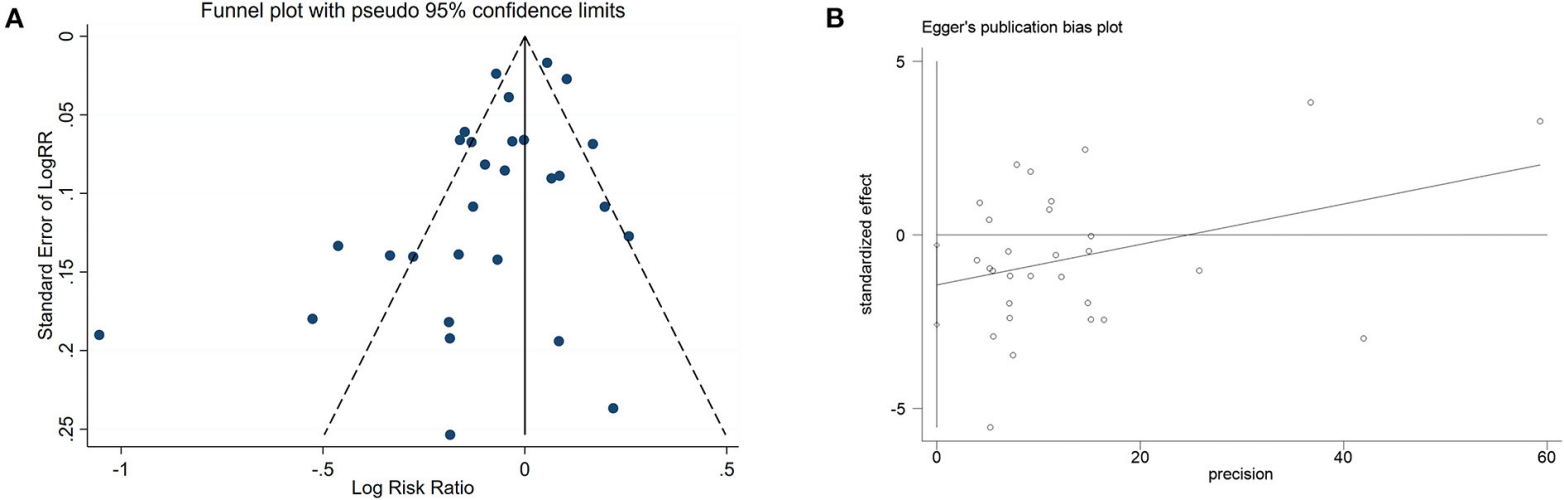
Funnel plot and Egger's test analyses to detect publication bias of studies on green tea consumption and coronary artery disease (CAD) risk. **(A)** Funnel plot: used to detect studies on green tea consumption and CAD risk publication bias. **(B)** Egger chart: used to detect studies evaluating green tea consumption and CAD risk publication bias.

A funnel plot (Figure 11) and Egger's test (Figure 11) were used to assess publication bias, with the result of Egger's test indicating that bias may exist. To determine the impact of this bias on the conclusion, we used the trim and fill method (diff = 0) and found that the final number of missing literature was k = 0, which indicated that despite the publication bias, our findings were robust.

**Figure 12:**
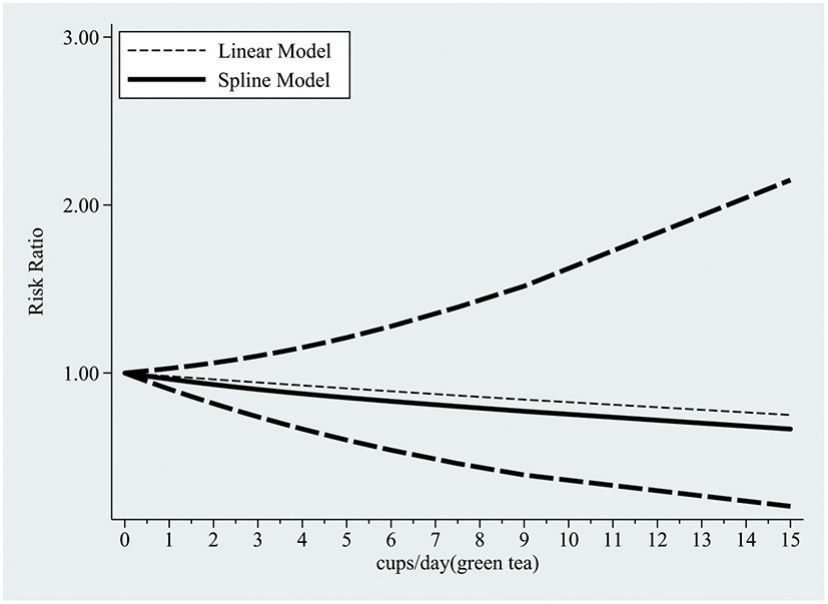
The dose-response relationship between green tea cup number (cups/d) and coronary artery disease (linear and non-linear models).

The dose-response analysis showed that the increase in green tea consumption by 1 cup per day did not result in a statistically significant reduction in the risk of CAD (summary RR = 0.98; 95% CI = 0.95, 1.02). However, the non-linear test result (I^2^ = 0.47%, *P* = 0.49) was statistically significant; thus, we fitted both non-linear and linear models (Figure 12). Our findings revealed that the RR of these two models decreased as the number of green tea cups consumed per day increased.

## Discussion

Tea is a traditional and popular beverage worldwide; regular tea consumption as part of a healthy habitual dietary pattern may substantially impact public health. To our knowledge, this is by far the largest meta-analysis assessing the association between tea consumption and its preventive effect on CAD. In this systematic review and meta-analysis of 35 observational studies, the pooled analysis showed that both black and green tea consumption might significantly reduce the risk of CAD. In dose-response analysis, a moderate amount of black tea drinking (<4 cups per day) has been shown to be beneficial; however, a large amount (more than 4–6 cups per day) has been shown to increase the risk of CAD. The dose-response relationship between green tea consumption and prevention of CAD based on linear and non-linear models revealed that the risk of CAD gradually decreased as green tea consumption increased.

The concept that tea intake may help prevent coronary artery disease has received much interest among medical professionals and the general public. The meta-analysis by Wang et al. (18) revealed that an increase in green tea consumption by one cup/d was associated with a 10% decreased risk of CAD. Consistent with findings from a meta-analysis by Wang et al. (18), we observed that green tea drinking could prevent CAD in this study. Concerning the biological mechanisms involved in the prevention of CAD, studies have shown that tea is high in antioxidant polyphenols (catechins, flavonols, theaflavins, and thearubigins) that protect the cardiovascular system (6, 57, 58). Catechins are flavan-3-ols that account for 30%–42% of the dry weight of tea (59). Studies have shown that catechin may promote nitric oxide production and enhance endothelial function (60, 61). Moreover, green tea contains more catechin polyphenols than black tea, and catechins have been shown to have vascular protective benefits through various pathways, including antioxidative, antihypertensive, anti-inflammatory, anti-proliferative, anti-thrombogenic, and lipid-lowering properties (62). Moderate tea consumption enhances endothelium-dependent vasodilation and significantly decreases blood pressure, which is beneficial for the cardiovascular system (63). Furthermore, tea drinking may reduce low-density lipoprotein cholesterol (LDL-c) and delay the formation of atherosclerosis (64). Tea's antioxidant and antithrombotic properties are significant in preventing CAD (62).

In line with our findings, a dose-response meta-analysis of 22 prospective observational studies (65) examining the relationship between tea consumption and major cardiovascular outcomes found that increasing tea consumption by three cups per day was associated with a lower risk of coronary heart disease, cardiac death, stroke, and cerebral infarction. However, the main limitation of the meta-analysis by Zhang et al. (65) was the use of diverse methodologies for evaluating tea intake and varied cup sizes. Another systematic review and meta-analysis (66) evaluating the link between tea consumption and CVD risks found that an increase in daily tea intake by one cup (236.6 mL) was associated with lower risks of CVD mortality by 4%. Nonetheless, in the present study increasing green tea intake by one cup per day did not result in a statistically significant decrease in the risk of CAD.

In contrast with the findings of a previous meta-analysis (18), we found that a moderate amount of black tea drinking (<4 cups/d) was associated with a reduction of CAD risk, while daily consumption exceeding 4–6 cups would increase the risk. To the best of our knowledge, the present study is the first to report these findings. As previously mentioned, catechins present in both green and black tea have cardioprotective qualities (60, 62). The catechin concentration of partly fermented black tea is about half that of green tea (67), which may explain the discrepancy in the dose-response relationship between black tea and green tea. Furthermore, a recent double-blind, randomized, placebo-controlled cross-over study (68) found that black tea may significantly raise central systolic blood pressure compared to green tea and placebo. Because black tea contains more caffeine than green tea, this effect could be attributed to caffeine. Therefore, we speculate that caffeine has a more significant impact on raising blood pressure and increases the risk of CAD when the dosage of black tea exceeds 4–6 cups. In subgroup analysis, we found a noteworthy and significant finding that there was a substantial inverse correlation between green tea drinking and the incidence of CAD in Asian populations but not in Western ones. Due to a lack of relevant gene sequencing data, we could not explain this variance from an ethnic or genetic viewpoint, although the influence of varied cooking techniques may play a role. Orientals prefer to boil tea with only hot water; however, in the west, high-lipid components such as milk are added to tea, which may elevate blood cholesterol and reduce the decrease the benefit of catechins (69, 70). In addition, Orientals treat tea drinking as a self-cultivation practice, and this emotional recognition may also affect blood pressure (68, 69).

### Strengths and limitations of this study

This study has the following strengths. Firstly, this systematic review and meta-analysis assessing the association between tea consumption and the risk of CAD are the largest to date, with more than two times the number of studies included in the previous meta-analysis. Secondly, we demonstrated a novel finding of a significant inverse association between green tea consumption and CAD risk in Asian populations but not in Western populations. Thirdly, we performed a dose-response analysis to investigate the linear and non-linear relationships, which may help quantify these possible associations' linkages. In addition, the majority of the studies included in this analysis are prospective studies, which would reduce the recall bias to a certain extent.

There are several limitations of this meta-analysis. Firstly, the quantity of antioxidant components such as flavonoids in tea varies depending on tea variety and region of origin, and the size of cups was not stated in detail in some research; therefore, we tried our best to avoid potential confounding bias from cup size. Secondly, this meta-analysis is based on observational studies, which are prone to bias. We cannot rule out uncontrolled confounders, such as fruit and vegetable consumption, socioeconomic status, and education level, as a possible explanations for the observed link between tea consumption and CAD risk. Thirdly, measurement mistakes in food and nutrient consumption estimations are unavoidable, and measurement errors may result in underestimating the relationship between tea consumption and CAD risk. Finally, all the articles included are in English, which may indirectly restrict the range of countries where the studies originated, so the findings may not be generalized to other areas of the world.

## Conclusions

In conclusion, the data presented in this study demonstrated that black tea and green tea had preventive effects on CAD. Green tea drinking could effectively reduce the risk of CAD in the Asian population (Chinese and Japanese), but not in Europeans and Americans. More than 4–6 cups of black tea consumption may promote CAD. The dose-response relationship of green tea showed that the risk of CAD gradually decreased with increased green tea consumption. Notably, the consumption of green tea in the Asian population is more popular than in western countries. Extrapolation of these results to the global population should be done with caution since the majority of studies included in the meta-analysis are from Asian nations, and few studies from other countries have been published. Therefore, further multi-center prospective studies should be conducted to examine the impact of tea drinking on the risk of CAD.

## Data availability statement

The original contributions presented in the study are included in the article/Supplementary material, further inquiries can be directed to the corresponding author/s.

## Author contributions

XY, HD, RD, MG, and JS: conception and design of the study. XY, ZZ, RD, and YQ: acquisition and analysis of the data. XY, HD, RD, and JS: interpreted the results. XY, HD, and ZZ: drafted the manuscript. XY, HD, RD, ZZ, MG, YQ, and JS: review and editing. All authors contributed to the article and approved the submitted version.

## Conflict of interest

The authors declare that the research was conducted in the absence of any commercial or financial relationships that could be construed as a potential conflict of interest. The handling editor declared a shared parent affiliation with the authors at the time of review.

## Publisher's note

All claims expressed in this article are solely those of the authors and do not necessarily represent those of their affiliated organizations, or those of the publisher, the editors and the reviewers. Any product that may be evaluated in this article, or claim that may be made by its manufacturer, is not guaranteed or endorsed by the publisher.
