# Supplementary material for: Association between tea consumption and prevention of coronary artery disease: A systematic review and dose-response meta-analysis
Source: Front Nutr. 2022 Nov 24;9:1021405. doi: 10.3389/fnut.2022.1021405 (PMC9729734; doi:10.3389/fnut.2022.1021405)
Supplement: Supplementary file 1 [file Data_Sheet_1.docx]

**Supplementary Material**

**Contents**

**Supplementary Appendix 1.** The complete search strategies employed in the PubMed database.

**Supplementary Table 1.** Quality assessments of included studies using the Newcastle-Ottawa scale.

**Supplementary Appendix 1- The complete search strategies employed in PubMed database**

**1. PubMed**

| ***Search ID*** | ***Search Terms*** |
| --- | --- |
| #1 | "Myocardial Ischemia" OR "myocard* infarct*" OR "coronary disease*" OR "ischemic heart" OR"atherosclerosis" OR "atherosclero*" OR "angina*" |
| #2 | ("tea" OR "black tea" OR "green tea" OR "flavonoid" OR "catechin" OR "cyanidanol" OR "theaflavin") AND ("Myocardial Ischemia" OR "myocard* infarct*" OR "coronary disease*" OR "ischemic heart" OR"atherosclerosis" OR "atherosclero*" OR "angina*") |
| #3 | #1 AND #2 |

**Supplementary Table 1.** Quality assessments of included studies using the Newcastle-Ottawa scale.

| **Reference** | **Types of tea** | **Selection** | | | | **Comparability** | **Outcome** | | | **Total** | **Overall assessment quality** |
| --- | --- | --- | --- | --- | --- | --- | --- | --- | --- | --- | --- |
|  |  | Representativeness  of exposed cohort | Selection of non-  exposed cohort | Ascertainment of exposure | Outcome present at start of study | Comparability of cohorts | Assessment of outcome | Length of  follow-up | Adequacy of  follow-up |  |  |
| Rosenberg et al (1980) | black tea | * | * | NR | * | * | * | * | NR | 6 | high-quality |
| Stensvold (1999) | black tea | * | * | * | * | * | NR | * | * | 7 | high-quality |
| De Koning Gans (2010) | black tea | * | * | NR | * | * | NR | * |  | 5 | low-quality |
| Geleijnse et al (2001) | black tea | NR | * | * | * | * | NR | NR | * | 5 | low-quality |
| Hertog et al （1993） | black tea | NR | * | * | * | * | NR | NR | * | 5 | low-quality |
| Hertog et al （1996） | black tea | * | * | * | * | * | NR | * | NR | 6 | high-quality |
| Keli et al  (1996) | black tea | NR |  | * | * | * | NR | * | NR | 4 | low-quality |
| Sesso et al（1999） | black tea | * | * | NR | * | NR | NR | * | NR | 4 | low-quality |
| Sesso et al（2003） | black tea | NR | * | * | * | * | NR | * | * | 6 | high-quality |
| Woodward et al (1999) | black tea | * | * | * | * | * | NR | * | * | 7 | high-quality |
| Klatsky et al (1993) | black tea | NR | * | * | * | * | NR | * | * | 6 | high-quality |
| Tavani et al (2002) | green tea | * | * | NR | * | * | NR | * | NR | 5 | low-quality |
| Thrift et al (1996) | green tea | * | * | NR | * | * | NR | * | NR | 5 | low-quality |
| Tian et al (2016) | green tea |  | * | NR | * | * | NR | * | NR | 4 | low-quality |
| Wang et al (2010) | green tea | * | * | NR | * | * | * | * | NR | 6 | high-quality |
| Hirvonen et al (2000) | green tea | NR | * | * | * | * | NR | * | * | 6 | high-quality |
| Wen et al (2008) | green tea | * | * |  | * | * | * | * |  | 6 | high-quality |
| Sato et al (1989) | green tea | * | * | * | * | * | NR | NR | * | 6 | high-quality |
| Hirano et al (2002) | green tea | * | * | NR | * | * | * | * | NR | 6 | high-quality |
| Gramenzi et al (1990) | green tea | * | * | NR | * | * | NR | * | NR | 5 | low-quality |
| Arthur et al (1999) | green tea | NR | * | * | * | * | NR | * | * | 6 | high-quality |
| Boston study (1972) | green tea | * | * | NR | * | * | NR | * | NR | 5 | low-quality |
| Hao et al (2015) | green tea | * | * | * | * | * | * | * | NR | 7 | high-quality |
| Sano et al (2014) | green tea | * | * | NR | * | * | NR | * | NR | 5 | low-quality |
| Miller et al (2016) | green tea | NR | * | NR | * | * | NR | * | NR | 4 | low-quality |
| Li et al (2017) | green tea | * | * | NR | * | * | NR | * | * | 6 | high-quality |
| Kishimoto et al (2020) | green tea | * | * | NR | * | * | * | * | NR | 6 | high-quality |
| Kokubo et al (2013) | green tea | * | * | NR | * | * | NR | * | * | 6 | high-quality |
| Pang et al (2016) | green tea | * | * | NR | * | * | NR | * |  | 5 | high-quality |
| Yan et al (2017) | green tea |  | * | * | * | * | NR | * | * | 6 | high-quality |
| Liu et al (2016) | green tea | * | * | * | * | * | NR | * | * | 7 | high-quality |
| Xiang et al (2018) | green tea | * | * | NR | * | * | * | * | NR | 6 | high-quality |
| Mineharu et al (2011) | green tea | * | * | NR | * | * | NR | * | NR | 5 | low-quality |
| Kuriyama et al (2006) | green tea | * | * | * | * | * | NR | * | NR | 6 | high-quality |
| Chen et al (2019) | green tea | * | * | NR | * | * | NR | * | NR | 5 | low-quality |

NR, not reported
